# Leaf Senescence by Magnesium Deficiency

**DOI:** 10.3390/plants4040756

**Published:** 2015-12-11

**Authors:** Keitaro Tanoi, Natsuko I. Kobayashi

**Affiliations:** Graduate School of Agricultural and Life Sciences, The University of Tokyo, 1-1-1 Yayoi, Bunkyo-ku, Tokyo 113-8657, Japan; E-Mail: anikoba@mail.ecc.u-tokyo.ac.jp

**Keywords:** leaf senescence, magnesium deficiency, transpiration

## Abstract

Magnesium ions (Mg^2+^) are the second most abundant cations in living plant cells, and they are involved in various functions, including photosynthesis, enzyme catalysis, and nucleic acid synthesis. Low availability of Mg^2+^ in an agricultural field leads to a decrease in yield, which follows the appearance of Mg-deficient symptoms such as chlorosis, necrotic spots on the leaves, and droop. During the last decade, a variety of physiological and molecular responses to Mg^2+^ deficiency that potentially link to leaf senescence have been recognized, allowing us to reconsider the mechanisms of Mg^2+^ deficiency. This review focuses on the current knowledge about the physiological responses to Mg^2+^ deficiency including a decline in transpiration, accumulation of sugars and starch in source leaves, change in redox states, increased oxidative stress, metabolite alterations, and a decline in photosynthetic activity. In addition, we refer to the molecular responses that are thought to be related to leaf senescence. With these current data, we give an overview of leaf senescence induced by Mg deficiency.

## 1. Introduction

Magnesium (Mg) is an essential macro-element for life, and it is usually present in cells as a divalent cation. Mg^2+^ plays an important role in many metabolic processes such as a cofactor of enzyme activity with ATP, a stabilizer for ribosomal structure, and the central atom in chlorophyll [1,2]. For steady metabolic processes, Mg homeostasis in the plant should be highly controlled. In support of this idea, Mg concentrations have the lowest variation among leaves of environmental samples [3] and the lowest variance among 18 minerals in *Arabidopsis* M3 mutants, based on an ionomic analysis [4].

**Figure 1:**
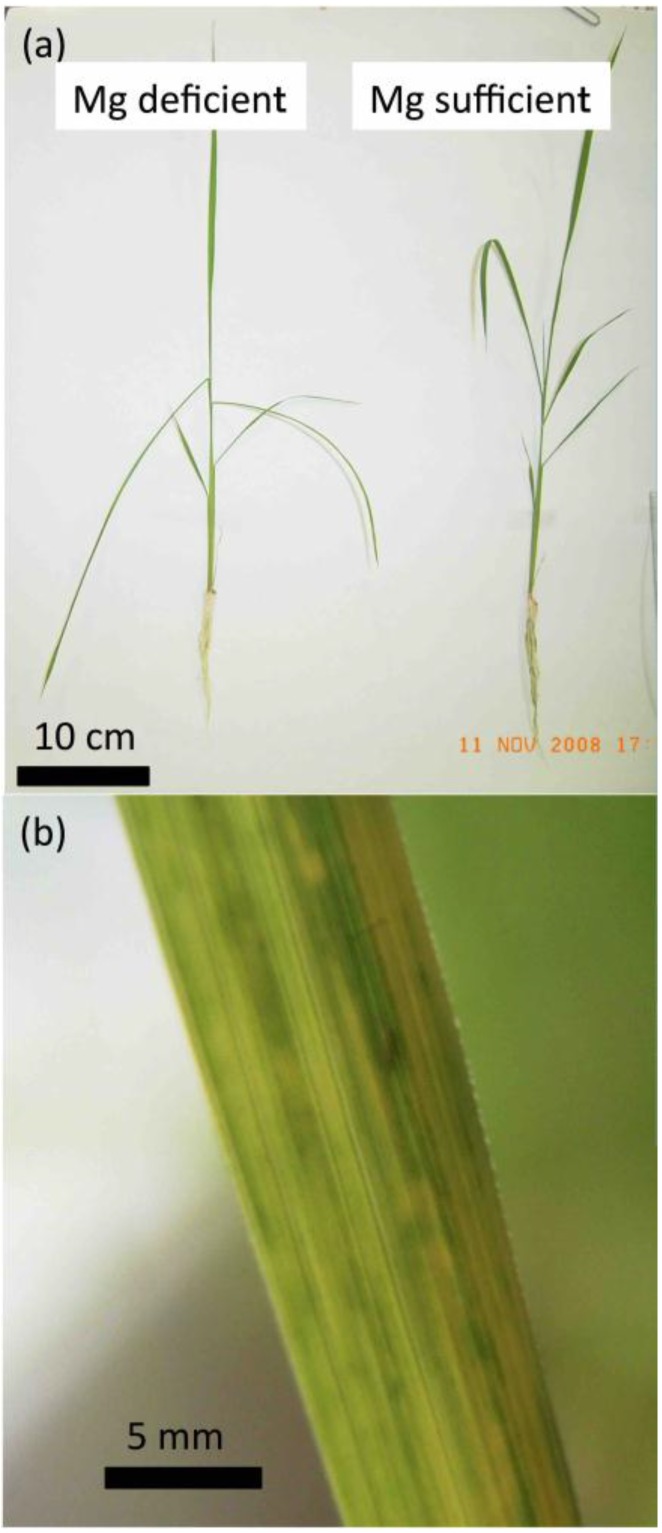

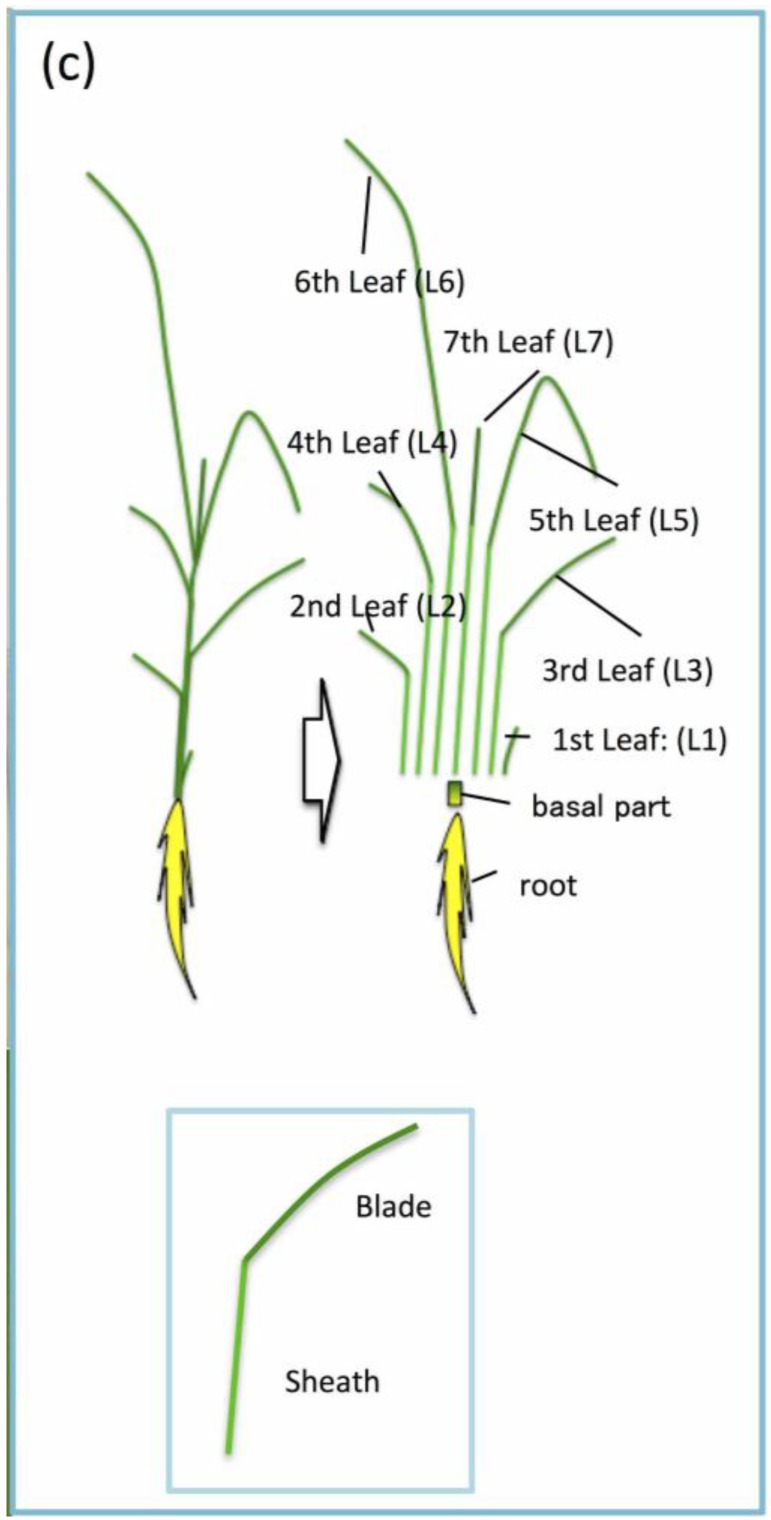
Typical symptoms of magnesium (Mg) deficiency in rice. (**a**) A rice plant cultured under Mg-deficient or Mg-sufficient conditions. The young mature leaves drooped under Mg deficiency. (**b**) Chlorosis of the leaf blade caused by Mg deficiency. (**c**) Schematic diagram of rice organs before tiller emergence. Leaf positions are numbered from oldest (L1) to newest (L7). Each leaf is composed of a leaf blade and leaf sheath except for L1.

Magnesium is abundant in the environment, with more than 20,000 ppm in the earth’s crust, making it the eighth most abundant element. Despite its abundance, Mg deficiency often occurs in the field because of soil acidity and competition with other cations such as hydrogen (H^+^), potassium (K^+^), calcium (Ca^2+^), and ammonium (NH_4_^+^) in the soil [5,6,7,8,9], especially in very sandy soils, and particularly in the culture of tobacco and potatoes [10]. The symptoms of Mg deficiency in crops have been pointed out by numerous researchers [8,9,11,12]. Under normal conditions, the Mg content is within the range of 0.15%–0.35% of the dry weight of the vegetative parts [2]. When the Mg content is below this range, symptoms emerge in the leaves. Symptoms tend to occur during the period of grain filling or fruit expansion, which influences the quality and quantity of the agricultural product [8,11,12]. Magnesium deficient leaves have yellowish, bronze, orange-yellow, or reddish tissues between the green veins [8,9,13], and sometimes they have brown interveinal necrosis (in rice; Figure 1). In general, mature leaves senesce faster than juvenile leaves when Mg availability is limited [9].

In the present review, we shall focus on the events that occur during leaf senescence under Mg deficiency. In addition, we shall mention the plant responses to adapt to the Mg deficient condition.

## 2. Alterations to Mg Distribution and the Potential Strategies to Recycle Mg during Deficiency

Experiments with hydroponic cultures have clarified the trends of Mg concentrations in roots and shoots. In the juvenile phase, the Mg concentration in the roots decreased within a couple of days after removing Mg from the solution, which was much faster than that in the shoots (e.g., in sugar beet [14], in *Arabidopsis* [15], and in rice [16]), indicating that Mg transport from root to shoot is not immediately downregulated by omitting Mg from the solution. Magnesium occurred at higher concentrations in the shoots than in the roots when Mg availability was sufficient (e.g., in sugar beet [17], in *Arabidopsis* [15], in rice [16,18], and in castor oil plant [19]), indicating a higher demand of Mg in the shoot. AtMHX, an Mg^2+^/H^+^ antiporter, localizes in the vacuolar membrane of xylem parenchyma cells [20] that engage in xylem loading in the roots and xylem unloading at sink organs [21]. Although information about AtMHX has been steadily provided, such as the regulation of expression by upstream open reading frames at translation levels [22], and the physiological characteristics of plants that overexpress the transporter [23], its function in long-distance transport remains unclear.

**Figure 2:**
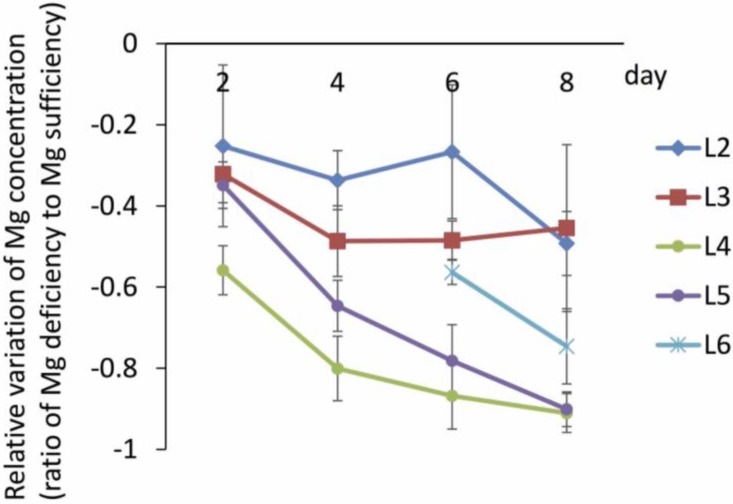
Time course of relative variation of magnesium (Mg) concentrations, modified from the data of Kobayashi *et al.* (2013) [16]. Values are shown as Mg concentrations in Mg-deficient leaves relative to control leaves. The seedlings of rice were grown hydroponically for two weeks and then transferred to a culture solution without Mg. The leaf positions are defined in Figure 1c.

Mg is a mobile ion in addition to nitrogen (N), phosphorus (P), and potassium (K) [24], and thus it is found at high concentrations (several mM) in the phloem [19,25]. This knowledge supports the observation that newer organs in the shoots have similar or higher Mg concentrations than older organs under Mg-limited conditions. However, when the differences in Mg concentrations between Mg-sufficient and -deficient plants were concerned, the bigger gap was found in young mature leaves (L4) rather than in old mature leaves (L2 and L3) of rice (Figure 2); similar trends were observed in sugar beet [17] and *Arabidopsis* [26,27]. This demonstrates that Mg retranslocation from old mature leaves is not as vigorous as that from young mature leaves. In contrast to Mg, translocation of K to the young sink organs from old mature leaves was reported to be comparable to that from young mature leaves under K-deficient conditions [28]. The reason for the difficulties in Mg retranslocation from old mature leaves remains unknown. Furthermore, the molecular mechanism of Mg translocation has not been characterized yet. Some Mg transporters have been shown to localize to vascular tissues [29], and thus might be involved in translocation. In the case of P, OsPT6, which is induced by P deficiency, is responsible for the retranslocation of phosphate in rice [30].

**Figure 3:**
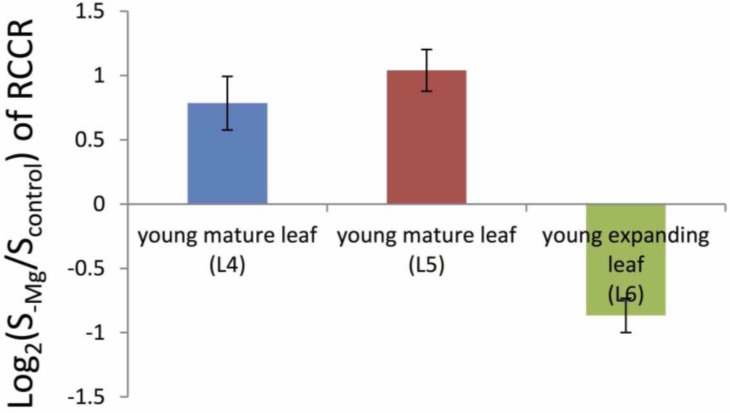
Alteration of red chlorophyll catabolite reductase (RCCR) expression in rice leaves under magnesium (Mg)-deficient conditions analyzed using microarray. Each leaf was sampled on Day 6 of an Mg-deficient treatment, after two weeks of pre-culture in the control solution. The leaf positions are defined in Figure 1c.

At the intracellular level, usually 6%–25% of the total Mg is bound to chlorophyll [2]. This proportion went up to more than 50% in Mg-deficient poplars grown under low light conditions [2]. This indicates the high requirement for Mg in chlorophyll and the high activity of Mg^2+^ transport into chloroplasts. The Mg transporters localized in the chloroplasts, AtMRS2-11/MGT10 and OsMRS2-6, are relatively well characterized and demonstrated to mediate Mg^2+^ transport [29,31,32,33,34]. Aside from the Mg partitioning in chlorophyll under Mg-deficient conditions, degradation of chlorophyll seems to be promoted by Mg deficiency. This is supported by transcriptomic studies showing the expression of genes related to the chlorophyll degradation processes, such as, non-yellowing 1 (NYE1: an initiator of chlorophyll degradation) in rice, multidrug resistance protein 3 (MRP3: a vacuolar ATP-binding cassette transporter that transports chlorophyll catabolites) in *Arabidopsis* [35,36], and red chlorophyll catabolite reductase (RCCR: a reductase involved in chlorophyll breakdown during senescence [37,38,39]) in rice (Figure 3). Degradation of chlorophyll might be related to the regulation of a “metabolic pool” in which Mg^2+^ concentrations are strictly regulated [2]. In addition to the intracellular processes, it is possible that Mg^2+^ release from chlorophyll is indirectly related to retranslocation. In the case of P, phosphatase activity was elevated by P deficiency or senescence, thereby changing the organic P in mature leaves to more mobile chemical forms, like inorganic P, which was then retranslocated to sink organs [40,41,42].

## 3. Initial Physiological Responses to Mg Deficiency

### 3.1. Young Mature Leaves are the Initial Sites of Mg-Deficiency Symptoms

**Figure 4:**
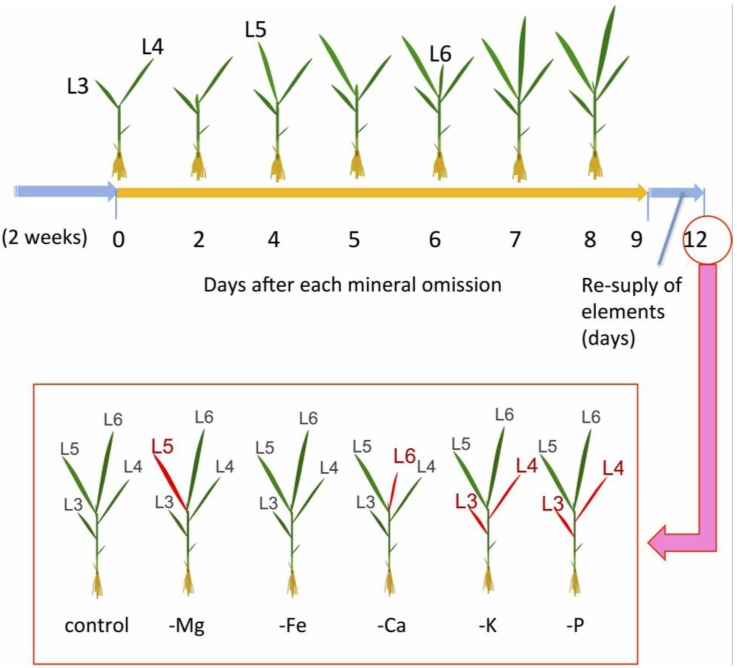
Experimental design for magnesium (Mg), iron (Fe), calcium (Ca), potassium (K), and phosphate (P) deficiency in rice. Seedlings were grown hydroponically for two weeks. The plants were transferred to culture solutions with the target mineral omitted and were grown for nine days. At Day 9, the plants were resupplied with each mineral. The plants were observed at Day 12. The red-colored leaf position indicates a dead leaf. The leaf positions are defined in Figure 1c.

Monocots are useful for observing every leaf position both separately and in detail. In rice, new leaves emerge about every two days, which makes it possible to determine the leaf maturing stage. Kobayashi *et al.* (2013) [16] performed an experiment whereby, for each nutrient element, a mineral deficiency was applied for nine days followed by mineral resupply (Figure 4). At Day 12, older leaves (L3 and L4) were dead in the P and K treatments, the newest leaf was dead and shoot growth had stopped in the Ca treatment (L6). At Day 12 in the Mg treatment, the L5 leaf that had experienced Mg-deficient conditions during the stages of rapid elongation, sink-source transition, and early maturation was dead but the other leaves were alive. Similar patterns of leaf senescence during Mg deficiency were observed in *Arabidopsis* and sugar beet [15,17]. Because the Mg deficiency status differs between the leaf positions, it is necessary to consider an experiment after identifying which leaf is the site of Mg deficiency before analyzing the mechanism of leaf senescence by Mg deficiency.

### 3.2. The Decline in Transpiration

The new mechanism of leaf senescence caused by Mg deficiency has been recently found. In rice, the leaf position L5 that began to emerge just after Mg deficiency started is the site of Mg deficiency, and it was the first leaf to senesce among all leaf positions (Figure 4). Several days before the photosynthetic rate declined in the sensitive leaf position, leaf transpiration rate decreased, which was measured by a portable photosynthesis system (LI-6400, LI-COR, Lincoln, NE, USA) with an attached leaf chamber [16]. Then, minerals in the xylem, including not only Mg^2+^ but also other solutes, were not supplied from the roots to the leaf [16]. Because the L5 had already expanded and become a source leaf, there was no influx flow via phloem to the leaf. Accordingly, the L5 leaf lost infrastructure for supply of water and minerals. Furthermore, it is hypothesized that the phloem flow from L5 is primarily stopped due to the lack of water supply, which might be the additional cause of the dysfunction of phloem loading frequently observed under the Mg deficiency (see Section 3.3). The symptom of decline in transpiration by Mg deficiency was also reported in citrus [43] and maize [44]. In addition, the resupply of Mg to Mg-deficient plants resulted in the recovery of transpiration [44].

The importance of the decline in transpiration was supported by the morphological change in Mg deficiency. The matured leaves were unable to stand up, instead they bent at the leaf collar and the blade shrank, indicating the loss of osmotic pressure of both the cells localized at the leaf collar, and the bulliform cells at the leaf blade [45], respectively. It is possible that the Mg concentration around the tissues might be too low for the cofactor of H^+^-ATPase to regulate the membrane potential, which can lead to dysfunction of holding appropriate osmotic pressure of the cells.

It is also possible that the decline in transpiration is directly related to drought stress. Preliminary results suggest that in rice, the transcription levels of OsMYB4, a transcription factor involved in cold acclimation and drought tolerance [46], and some of the OsDREBs, transcription factors involved in drought, high salt, and cold stress [47], were induced by Mg-deficient leaves. Further experiments are needed to clarify the mechanisms involved.

### 3.3. Sugar and Starch Accumulation

Photosynthates and starches accumulate in young mature leaves before any decline in biomass or photosynthetic activities in common bean [48,49,50], sugar beet [14,17], and *Arabidopsis* [15]. Potassium deficiency induces soluble sugar accumulation in the leaves but without starch accumulation in common bean [49], *Arabidopsis* [51], sugar beet [36], rice [16,18,52], and citrus [53].

Sucrose accumulation in source leaves was explained by the defect of phloem loading of sucrose [50]. Phloem dysfunction was suggested to be caused by the mechanism whereby the lack of Mg reduces the activity of H^+^-ATPase localized at sieve tube membranes, leading to the depolarization of the membrane potential, and the decline in phloem loading of sucrose through sucrose-H^+^ symporters [36,54]. The elevated transcript level of the sucrose/H^+^ transporter gene *BvSUT1* might indicate that plants try to cope with the increased sucrose content by Mg deficiency [17]. When Mg was resupplied to plants accumulating sucrose by Mg deficiency, the sucrose level decreased as early as 12 h afterward [50], clearly demonstrating the importance of Mg on phloem loading of sucrose.

Starch accumulation in chloroplast at source leaves is one of the typical symptoms of Mg deficiency. The key enzymes of starch synthesis such as ADP-glucose pyrophosphorylase [55,56] and phosphoglucomutase [57] localize in chloroplast and demand Mg^2+^ as a cofactor. It indicates that the Mg concentration in chloroplasts remains adequate even though the cells around sieve elements are struggling with low Mg condition and the phloem loading of sucrose looses its function. Verbruggen and Hermans (2013) [36] also discussed that the potential prime targets of Mg depletion are the sieve elements and companion cells rather than the chloroplasts of mesophyll parenchyma. Nevertheless, the regulation system of starch biosynthesis in source leaves remains unclear even under Mg sufficient conditions [56]. There have been reports showing the relationship between sucrose synthases (SuSy) and starch accumulation in tomato stems [58] whereby SuSy controlled the starch biosynthesis process in potato source leaves [59,60]. On the other hand, SuSy was not essential for normal growth or normal amounts of leaf starch synthesis in *Arabidopsis* [61]. The mechanism of starch accumulation induced by Mg deficiency is far from being understood.

The accumulation of carbohydrates in source leaves induced by Mg deficiency causes alterations in photosynthetic carbon metabolism and restricts CO_2_ fixation, probably leading to the generation of reactive oxygen species (ROS) through leakage of electrons and absorbed energy from photosynthesis [62]. Indeed, the starch-accumulating site visualized by iodine staining seemed to coincide with the area of chlorotic symptoms in *Arabidopsis* [15], sugar beet [17], and rice [16].

### 3.4. The Influence on the Photosynthetic Apparatus by Mg-Deficiency Associated with Chlorophyll

After sugars and starches begin to accumulate, a decline in photosynthetic activity appears. Recently, the events relating to a decline in photosynthesis by Mg deficiency were described well in sugar beet [14] and in *Sulla carnosa* [63]. In sugar beet, the photosystems (PS) II and I were downregulated by Mg deficiency, although with contrasting responses. The light absorption processes relating to antenna complexes containing Chl *b* were reduced in PSII. On the other hand, the amount of core complexes were reduced in PSI [14]. In *Sulla carnosa*, low Mg condition caused negative effects on the abundance of PSI-related polypeptides, and consequently PSI photochemistry and capacity for cyclic electron transport, showing the main target of Mg deficiency is PSI rather than PSII [63]. In addition, a proteomic analysis using matrix-assisted laser desorption/ionization time-of-flight tandem mass spectrometry on Mg-deficient citrus demonstrated the decrease of many proteins involved in photosynthesis [43]. The subsequent influence on chlorophyll synthesis and chlorophyll *a/b* ratio was well documented in the review by Verbruggen and Hermans (2013) [36].

## 4. The Mechanism of Chlorosis and Necrosis of Leaves Induced by Mg Deficiency

The candidates causing chlorosis and necrosis by Mg deficiency were reported to be photosynthate accumulation, ROS, redox change, abscisic acid (ABA) signaling, ethylene response, and metal accumulation. In general, the combination of ROS and antioxidative defenses is associated with any kind of stress reaction, including Mg deficiency. Cakmak and Kirkby (2008) [62] found that the accumulation of carbohydrates and the impairment in photosynthetic CO_2_ fixation in Mg-deficient leaves appears to proceed to ROS generation. Peng *et al.* (2015) [43] reported that enhancing the levels of proteins involved in ROS scavenging, such as superoxide dismutase (SOD), was found both in the roots and leaves of citrus under Mg deficiency. Anthocyanins, which could be involved in photoprotection as an antioxidant [64], accumulated in the source leaves during Mg-deficiency in rice, suggesting ROS-mediated leaf senescence [16]. Mg-deficiency induced ROS scavengers such as reduced ascorbic acid and SH-containing compounds [62,65], as well as the activities of SOD [65,66], ascorbate peroxidase [65], glutathione reductase [65,66], and catalase [65]. In addition, Mg deficiency changed the redox balance to more reduced forms of ascorbate [35] and glutathione [35,65,66], suggesting that the protection against oxidative stresses was caused by Mg deficiency.

Mg deficiency influences plant hormone levels and signaling. Ethylene, a plant hormone responsible for fruit ripening, leaf senescence, and stress response signaling, was reported to be increased by Mg deficiency [35]. Also, the genes encoding ethylene biosynthetic enzymes were enhanced in *Arabidopsis* [35], and in rice (our recent data). Interestingly, ethylene production was increased under mild drying in tomato leaves [67,68]. Furthermore, ABA signaling might be one of the main targets of Mg deficiency. Hermans *et al.* (2010) [26] showed that half of the upregulated genes in leaves with short-term Mg-deficiency were also ABA-responsive in *Arabidopsis*. Interestingly, the central oscillator of the circadian clock in roots was also influenced by short-term Mg-deficiency [26], which might be involved in a part of ABA and ethylene signaling that is dependent on the circadian clock or circadian rhythm [36].

ABA and metabolites related to water stress such as citrate and *myo*-inositol were influenced by Mg deficiency in rice [16]. Citrate accumulation occurred with drought and salinity stress [69,70,71,72] as well as with ABA treatment [73]. *Myo*-inositol derivatives were present as lipids and water-soluble compounds, and their metabolism is related to water stress [74]. The concentration of *myo*-inositol in sensitive leaf positions of rice decreased by 50% from that of the control during Mg deficiency, and it did not show any decline during K, iron (Fe), Ca, and P deficiency [16]. The large decreasing rate, 50%, implied an important role for *myo*-inositol signaling in response to Mg deficiency. Interestingly, the influences on *myo*-inositol concentration and citrate were found before any symptoms emerged, except for the decline in transpiration in rice leaves sensitive to low Mg [16].

**Figure 5:**
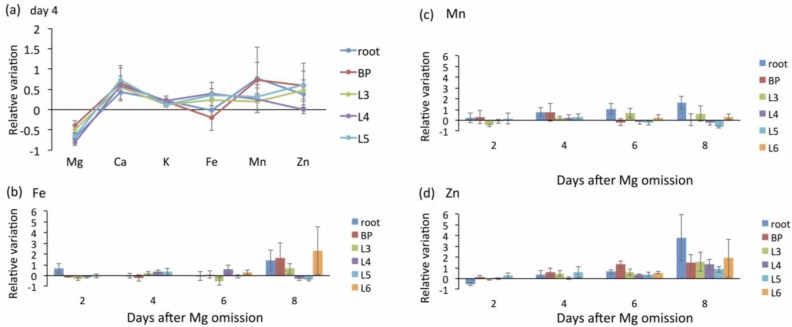
Relative variations of minerals. (**a**) The relative mineral concentrations under magnesium (Mg) deficiency at Day 4. The leaf positions (L3, L4, L5, and L6) and other organs (root and basal part (BP)) are defined in Figure 1c. (**b**) Time course of relative iron (Fe) concentrations in Mg-deficient plants. (**c**) Time course of relative manganese (Mn) concentrations in Mg-deficient plants. (**d**) Time course of relative zinc (Zn) concentrations in Mg-deficient plants.

By employing a multi-element analysis technique, the ion balance under Mg deficiency was examined in *Arabidopsis* [27,35] and in rice (Figure 5). In *Arabidopsis*, the concentrations of Ca, Fe, and zinc (Zn) in young mature leaves were increased by Mg deficiency [35], which was similar to the effects observed in rice. Similarly, the concentrations of manganese in young leaves were tripled by Mg deficiency in mulberry [75]. It is possible that the imbalance of minerals causes ROS production [35].

## 5. Responses of Mg Transporters to Mg Deficiency

**Figure 6:**
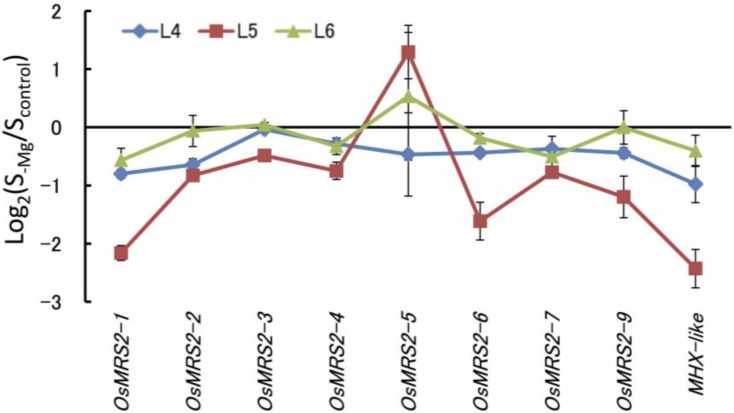
Relative expression of magnesium (Mg) transporters in each rice leaf position. Each leaf was collected and analyzed at Day 6 of the Mg-deficient condition following two weeks of culture with the control solution. The leaf positions (L4, L5, L6) are defined in Figure 1c.

The activities of transport or translocation systems are dependent on the availability of most essential minerals (e.g., phosphate [76], nitrate [77], ammonium [78], potassium [79], sulfate [80], iron [81,82], manganese [83], zinc [82,84] and boron [85]). In the bacterium *Salmonella*, expression of the transport systems MgtA and MgtB are transcriptionally induced by low Mg conditions [86] and are regulated by the Mg^2+^-regulated PhoP-PhoQ two-component system [87,88,89]. Recently, Mao *et al.* (2014) [90] reported that the gene *MGT6/MRS2-4* was induced by Mg deficiency as early as 12 h afterward and that it contributed to Mg uptake under low Mg conditions. In contrast, gene expression of the MRS2 family of transporters was reported to have no response to short- or long-term Mg-deficiency by transcriptomic studies [26,35], and most of the homologous genes of rice were downregulated by Mg deficiency (Figure 6). Further study is needed to clarify this contradiction. Regarding the physiological responses, Mg uptake activity assayed using ^28^Mg^2+^ was elevated under Mg deficiency in rice, which was drastically reduced almost to the control level or slightly more within 5 min after Mg resupply. The similar mechanism of the structure modification at the gating site for cytoplasmic Mg2+ reported in MgtE [91] and CorA [92,93] might be involved in the rapid response in plant roots.

## 6. Summary and Perspective

**Figure 7:**
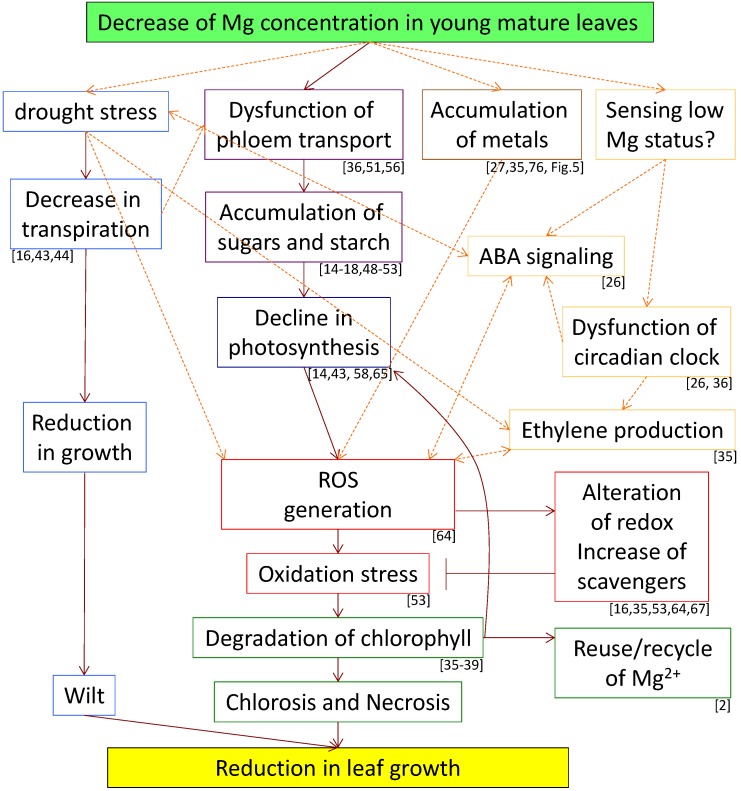
Schematic summary of events and their interrelationships in young mature leaves under magnesium (Mg) deficient conditions. The colors of frame borders shows categories; light blue: drought stress, purple: accumulation of photosynthates, brown: accumulation of metals, yellow: hypothetic signaling, blue: photosynthesis, red: oxidation stress, green: chlorophyll. The orange dotted lines indicate the hypotheses shown by recent studies and the present review. ABA, abscisic acid; ROS, reactive oxygen species.

After many years of research examining the mechanism of Mg deficiency, the overall hierarchy of plant responses to Mg deficiency has been clarified (Figure 7). However, the studies that reveal the regulatory network of the senescence symptoms of Mg deficiency have been just started. In particular, the involvement of ABA and ethylene signaling in the regulatory network are of interest for further studies. A combination of omics approaches, such as transcriptomics [26,35], proteomics [43,94], and ionomics [27], would be useful for clarifying the molecular regulatory system. Surprisingly, there is only a single report employing forward genetics to examine Mg deficiency [95]. Definitive evidence is expected to be provided by mutants obtained using forward genetics as well as reverse genetics in future investigations.

Plants have diverse responses to Mg deficiency, most of which are related to coping with the problems occurring upon Mg absence, such as chlorophyll degradation and antioxidation. In addition, the fact that there are various levels of tolerance to Mg deficiency among plants makes us realize that veiled mechanisms of Mg tolerance must exist in the plant kingdom. In this context, it is worth noting the importance of dealing with plants tolerant to Mg shortage. To resolve the Mg-deficiency problem in the agricultural field, we require more knowledge about the mechanisms of plant senescence caused by conditions of low Mg.
